# Lattice plainification and band engineering lead to high thermoelectric cooling and power generation in n-type Bi_2_Te_3_ with mass production

**DOI:** 10.1093/nsr/nwae448

**Published:** 2024-12-06

**Authors:** Dongrui Liu, Shulin Bai, Yi Wen, Jiayi Peng, Shibo Liu, Haonan Shi, Yichen Li, Tao Hong, Huiqiang Liang, Yongxin Qin, Lizhong Su, Xin Qian, Dongyang Wang, Xiang Gao, Zhihai Ding, Qian Cao, Qing Tan, Bingchao Qin, Li-Dong Zhao

**Affiliations:** School of Materials Science and Engineering, Beihang University, Beijing 100191, China; Center for Bioinspired Science and Technology, Hangzhou International Innovation Institute, Beihang University, Hangzhou 311115, China; School of Materials Science and Engineering, Beihang University, Beijing 100191, China; School of Materials Science and Engineering, Beihang University, Beijing 100191, China; School of Materials Science and Engineering, Beihang University, Beijing 100191, China; School of Materials Science and Engineering, Beihang University, Beijing 100191, China; School of Materials Science and Engineering, Beihang University, Beijing 100191, China; School of Materials Science and Engineering, Beihang University, Beijing 100191, China; School of Materials Science and Engineering, Beihang University, Beijing 100191, China; Hebei Key Laboratory of Optic-Electronic Information and Materials, College of Physics Science and Technology, Hebei University, Baoding 071002, China; School of Materials Science and Engineering, Beihang University, Beijing 100191, China; School of Materials Science and Engineering, Taiyuan University of Science and Technology, Taiyuan 030024, China; Hebei Key Laboratory of Optic-Electronic Information and Materials, College of Physics Science and Technology, Hebei University, Baoding 071002, China; Key Laboratory of Materials Physics of Ministry of Education School of Physics, Zhengzhou University, Zhengzhou 450001, China; Center for High Pressure Science and Technology Advanced Research (HPSTAR), Beijing 100094, China; Huabei Cooling Device Co. LTD, Langfang 065400, China; Huabei Cooling Device Co. LTD, Langfang 065400, China; School of Materials Science and Engineering, Beihang University, Beijing 100191, China; School of Materials Science and Engineering, Beihang University, Beijing 100191, China; Center for Bioinspired Science and Technology, Hangzhou International Innovation Institute, Beihang University, Hangzhou 311115, China; School of Materials Science and Engineering, Beihang University, Beijing 100191, China; Center for Bioinspired Science and Technology, Hangzhou International Innovation Institute, Beihang University, Hangzhou 311115, China; Tianmushan Laboratory, Hangzhou 311115, China

**Keywords:** thermoelectric, n-type Bi_2_Te_3_, lattice plainification, carrier mobility, thermoelectric device

## Abstract

Thermoelectrics can mutually convert between thermal and electrical energy, ensuring its utilization in both power generation and solid-state cooling. Bi_2_Te_3_ exhibits promising room-temperature performance, making it the sole commercially available thermoelectrics to date. Guided by the lattice plainification strategy, we introduce trace amounts of Cu into n-type Bi_2_(Te, Se)_3_ (BTS) to occupy Bi vacancies, thereby simultaneously weakening defect scattering and modulating the electronic bands. Meanwhile, the interstitial Cu can bond with the BTS matrix to form extra electron transport pathways. The multiple occupations of Cu substantially boost carrier mobility and electrical performance. Consequently, the BTS + 0.2%Cu achieves a room-temperature *ZT* of ∼1.3 with an average *ZT*_ave_ of ∼1.2 at 300–523 K. Moreover, the kilogram-scale ingot designed for mass production also exhibits high uniformity. Finally, we fabricate a full-scale device that achieves an excellent conversion efficiency of ∼6.4% and a high cooling Δ*T*_max_ of ∼70.1 K, both of which outperform commercial devices.

## Teaser

Incorporating trace amounts of Cu atoms into n-type Bi_2_(Te, Se)_3_ simultaneously realizes lattice plainification and band structure engineering, thus substantially facilitating thermoelectric transport and leading to excellent device efficiencies.

## INTRODUCTION

As the global energy crisis intensifies, the depletion of traditional energy resources and environmental pollution issues have become increasingly severe [1]. This compels researchers to accelerate the search and development of more efficient and environmentally friendly energy technologies [2,3]. In this context, thermoelectric technology, which utilizes the Seebeck and Peltier effects to achieve efficient energy conversion, has become an important research direction in the current scientific field [4]. The thermoelectric performance is assessed by the dimensionless figure of merit *ZT*, defined as *ZT* = (*S*²*σ*)*T*/(*κ*_ele_ + *κ*_lat_ + *κ*_bip_), where *σ* represents electrical conductivity, *S* is the Seebeck coefficient, *T* is the Kelvin temperature, and *κ*_ele_, *κ*_lat_, and *κ*_bip_ denote the electronic, lattice, and bipolar thermal conductivity, respectively [5]. To decouple the intrinsically correlated phonon-carrier transport and thus improve thermoelectric performance, numerous methods and strategies have been established, representatively including tuning carrier concentration [3,6–8], enhancing carrier mobility [9–12], engineering electronic band structures [13–15], constructing all-scale microstructures [16–21], and searching novel systems with intrinsic low thermal transport [20,22–24].

Bismuth telluride (Bi₂Te₃) is one of the few thermoelectrics that can achieve a *ZT* value of up to ∼1.0 near room temperature [25]. It includes both p-type and n-type thermoelectrics [26], making it an excellent candidate for large-scale commercial power generation and cooling applications [27]. However, due to the intrinsic narrow bandgap of Bi_2_Te_3_, a large number of minority carriers are excited at high temperatures, leading to a significant increase in *κ*_bip_ and a substantial decrease in *S*, which ultimately results in a notable deterioration of thermoelectric performance at above ∼400 K and limits their application in low-grade waste heat recovery [28]. Increasing carrier concentration is an effective strategy to suppress bipolar diffusion, but it also worsens the room-temperature thermoelectric performance [29]. How to adopt effective optimization strategies to balance the thermoelectric performance of Bi_2_Te_3_ at both room- and mid-temperatures, thereby achieving an excellent average *ZT* across a wide temperature range, is key to advancing the application of Bi₂Te₃-based devices [30].

In recent years, researchers have successfully stabilized the *ZT* value of p-type (Bi, Sb)₂Te₃ (BST) above ∼1.0 by introducing second-phase nanostructures or all-scale hierarchical architectures to finely regulate the microstructures [31–33]. However, to achieve high conversion efficiency of thermoelectric devices, it is crucial to attain a *ZT* value for n-type counterparts comparable to that of p-type thermoelectrics [34]. Although numerous studies have optimized n-type Bi_2_Te_3_ by microstructure design, the corresponding improvement in *ZT* values has been limited [35,36]. This is primarily due to the inherent stronger anisotropy of n-type Bi₂Te₃, which makes its electrical properties highly sensitive to orientation [37]. The powder metallurgy processes commonly used to introduce nanostructures can lead to a random distribution of grains within the lattice, thereby reducing the preferred orientation and leading to deterioration of electrical performance [37]. Therefore, the texture process that achieves high orientation is more effective for optimizing the thermoelectric performance of n-type Bi₂Te₃ [38,39]. For instance, hot extrusion, hot forging, and liquid-phase hot deformation techniques have proven to be effective in facilitating grain rearrangement, resulting in enhanced orientation and superior thermoelectric performance along the (00*l*) direction [40]. However, these techniques have been facing numerous challenges in practical mass production including severe process parameters such as temperature and pressure at every stage and possible variations in performance between different production batches, making them not as stable and reliable as the zone melting (ZM) method for being used in commercial production [35]. Therefore, this study aims to optimize the composition of commercial n-type Bi_2_Te_3_ mass-production ingots produced by the ZM technology to further enhance the efficiency of commercial devices.

Recently, the lattice plainification strategy has proven to be rather effective in optimizing the low- to mid-temperatures, by precisely tuning the lattice defects in thermoelectrics [12,41,42]. Bi_2_Te_3_-based thermoelectrics are widely considered to have numerous intrinsic defects such as anion and cation vacancies and anti-site defects, due to the close electronegativity of Bi and Te [43]. This opens up ample possibilities for us to implement the lattice plainification strategy in this system. In this study, active Cu is selected for incorporation into the commercial n-type I-doped Bi₂(Te, Se)₃ (abbreviated as BTS) ingots. Unlike previous research that enhances thermoelectric performance by introducing substantial amounts of Cu to regulate carrier concentration [34,44], this study employs only trace amounts of Cu for defects manipulation.

**Figure 1.**
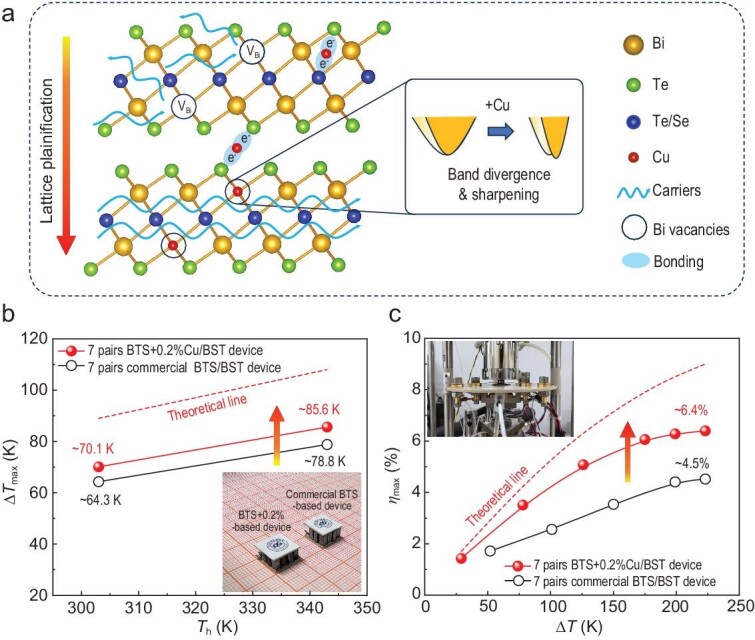
(a) The schematic diagram illustrates the optimization mechanism for the n-type BTS + Cu in this study. Cu can fill Bi vacancies to weaken defect scattering and adjust the band structure. Furthermore, interstitial Cu can bond with adjacent atoms to create extra charge transport channels, thereby optimizing carrier mobility synergistically. (b) Maximum cooling temperature difference Δ*T*_max_ for the BTS + 0.2%Cu/BST-based device and the commercial BTS/BST-based device. The red dashed line represents the theoretical cooling Δ*T*_max_ for the BTS + 0.2%Cu/BST-based device. The inset shows a photograph of the two thermoelectric devices. (c) Maximum conversion efficiency *η*_max_ for the BTS + 0.2%Cu/BST-based device and the commercial BTS/BST-based device. The red dashed line represents the theoretical *η*_max_ for the BTS + 0.2%Cu/BST-based device. The inset shows a Mini-PEM instrument test image of the thermoelectric device.

As shown in Fig. 1a, the small amount of Cu leads to several significant effects: Cu can occupy intrinsic Bi vacancies to achieve lattice plainification [42], thereby weakening the point defect scattering for carriers. The Cu occupying the Bi site also modifies the electronic band structure to promote conduction band divergence and sharpening, which reduces the effective mass m* and further enhances *μ*. Additionally, Cu atoms located in the van der Waals (vdW) gaps and the quintuple layers (-Te-Bi-Te-Bi-Te-) as interstitials are found to be bonded with neighboring atoms, forming extra electron transport channels to further improve *μ*. Collaborative optimization by multiple occupations of Cu has resulted in an outstanding *μ* of ∼285 cm² V^−^¹ s^−^¹ and an ultrahigh power factor (*PF*) of ∼60 μW cm^−^¹ K^−^² for the BTS + 0.2%Cu ingot at 300 K. Ultimately, the *ZT* value approaches ∼1.3 at 300 K, with a high average *ZT* (*ZT*_ave_) of ∼1.2 at 300–523 K. Furthermore, the 7-pair thermoelectric device, utilizing the optimized n-type BTS + 0.2%Cu in conjunction with commercial p-type (Bi, Sb)_2_Te_3_ (BST), exhibits a maximum cooling temperature difference (Δ*T*_max_) of ∼70.1 K at a hot-side temperature (*T*_h_) of ∼303 K. As *T*_h_ increases to ∼343 K, Δ*T*_max_ rises to ∼85.6 K (Fig. 1b). These cooling efficiencies are much larger than the commercial BTS/BST-based device. For power generation, the as-fabricated 7-pair thermoelectric device exhibits a maximum conversion efficiency *η*_max_ of ∼6.4% at a temperature difference (Δ*T*) of ∼223 K, which significantly outperforms the commercial device with a *η*_max_ of ∼4.5% (Fig. 1c). In addition to excellent thermoelectric performance, the kilogram-scale BTS + Cu ingot with mass production exhibits good performance uniformity and mechanical processing performance over the entire sample, making it highly suitable for being directly utilized in electronic cooling and waste heat recovery.

## RESULT AND DISCUSSION

### Electrical performance of n-type Cu-doped BTS

**Figure 2.**
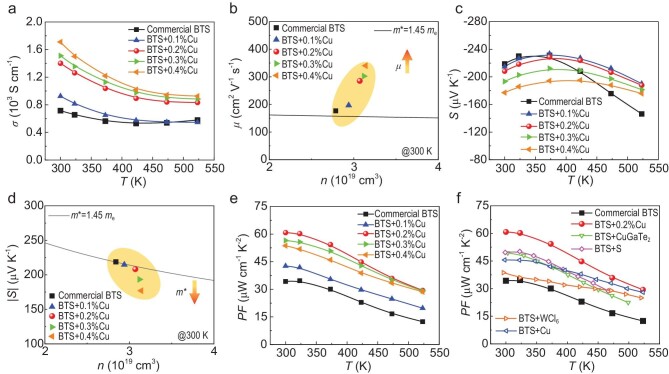
Electrical transport properties of BTS + x%Cu samples (x = 0, 0.1, 0.2, 0.3, and 0.4). (a) Electrical conductivity *σ*. (b) The carrier mobility *μ* as a function of carrier concentration *n*. (c) Seebeck coefficient *S*. (d) The absolute value of Seebeck coefficient |*S*| as a function of carrier concentration *n*. (e) Power factor *PF*. (f) Comparison of *PF* curves between the BTS + 0.2%Cu samples and other high-performance n-type BTS [35,39,40,45].

We prepared a series of BTS + x%Cu (x = 0, 0.1, 0.2, 0.3, and 0.4) samples using a zone melting method and investigated their transport properties along the crystal growth direction, since the thermoelectric performance of the commercial BTS + 0%Cu sample along the in-plane and out-of-plane directions shown in Fig. S1 indicates the anisotropic transport properties of the BTS system, and the in-plane direction (crystal growth direction) demonstrates better performance. Figure 2a illustrates the temperature-dependent electrical conductivity *σ* for BTS + Cu samples. With increasing Cu content, *σ* rises from ∼715 S cm^−^¹ for commercial BTS to ∼1710 S cm^−^¹ for BTS + 0.4%Cu at 300 K. To gain further insight into the *σ* variation, we plotted the relationship between *n* and *μ* at 300 K, as depicted in Fig. 2b. The *n* increases with Cu content, while the rate of increase diminishes after x = 0.2. This gradual rise in *n* was attributed to interstitial Cu atoms acting as n-type dopants. The deceleration in the rate of increase was due to the fact that, once interstitial sites were filled to some extent, additional Cu atoms started to occupy Bi sites, acting as p-type dopants and thereby increasing the hole concentration. Additionally, as shown in Fig. 2b, the *μ* increased with higher Cu content. This enhancement occurs because Cu fills the Bi vacancies, weakening point defect scattering, while also adjusting the band structure to reduce m*, thereby collectively improving *μ*. At the same time, interstitial Cu atoms can bond with neighboring atoms to form additional charge transport pathways, resulting in a further increase in *μ*. Besides, Fig. S2 shows that all Cu-doped BTS samples have a lower deformation potential compared to BTS + 0%Cu. Among them, BTS + 0.2%Cu exhibits the lowest deformation potential, indicating that the introduction of Cu effectively decouples electron-phonon transport. Figure 2c illustrates the variation of *S* with temperature. As *S* was inversely related to *n*, its value decreased gradually with increasing Cu content at room temperature. Furthermore, the peak of *S* shifted to higher temperatures, which could be attributed to the increased *n* delaying the intrinsic thermal excitation. Figure 2d illustrates the relationship between |*S*| and *n*. It was evident that beyond x = 0.2, m* decreased significantly, suggesting that Cu atoms began to alter the band structure, consistent with the trend in *μ* presented in Fig. 2b. Ultimately, the boosted *μ* enabled the BTS + 0.2%Cu sample to achieve an outstanding *PF* value of ∼60 µW cm^−1^ K^−^² at 300 K (Fig. 2e), nearly double when compared to the Cu-free sample and other high-performance n-type BSTs (Fig. 2f) [35,39,40,45].

### Complex occupation of Cu atoms in the lattice

Figure S3a shows the X-ray diffraction (XRD) patterns of BTS + x%Cu (x = 0, 0.1, 0.2, 0.3, and 0.4) samples at room temperature. The diffraction peaks were indexed to the Bi_2_Te_3_ phase with the R3̅m space group (JCPDS#15–0863). Moreover, no diffraction peaks corresponding to impurity or secondary phases were detected in any of the diffraction patterns, confirming the complete dissolution of Cu in the lattice. The Cu atoms significantly affected the lattice constant of BTS alloys. It could be observed that, with increasing Cu content, the angle of the main diffraction peak exhibited a decreasing, increasing, and then decreasing trend, indicating that the occupation of Cu in the lattice may not be unique.

Lattice parameters calculated from Rietveld refinement (Fig. S3b) revealed that both the lattice parameters of *a*-axis and *c*-axis exhibited similar trends with increasing Cu content. At a Cu concentration of 0.2% mol, the lattice parameters initially decreased sharply and then gradually increased. If Cu atoms were only to occupy the van der Waals (vdW) gaps, as indicated in previous studies [7], the *a*-axis lattice parameter would remain relatively unchanged while that of the *c*-axis would increase steadily. However, the experimental results of this study were contrary to previous studies, suggesting that Cu occupies more than just the vdW gaps. Additionally, by combining the trends in carrier concentration *n* for BTS + x%Cu samples (Fig. 2b), *n* initially increased rapidly and then gradually slowed down. Therefore, it could be inferred that Cu may simultaneously exhibit both p-type and n-type doping, and based on this, a comprehensive analysis of the behavior of Cu sites in the lattice could be conducted.

After entering the lattice, Cu first acted as an n-type dopant, occupying two different interstitial sites: between the vdW gaps and inside the quintuple layers consisting of five covalently bonded atomic planes (-Te-Bi-Te-Bi-Te-). This caused the increased lattice parameters. These two types of interstitial sites for Cu have been confirmed by the microstructure characterization as will be discussed later. When the Cu doping content was 0.2% mol, Cu atoms started to substitute Bi, which could be considered as p-type doping. And, the p-type doping behavior of Cu has been confirmed by the subsequent defect formation energy and was also consistent with the slowly increasing trend of *n* shown in Fig. 2b. Additionally, Cu substituted Bi, leading to a decrease in the lattice parameters, due to the smaller ionic radius of Cu²⁺ (∼72 pm) compared to that of Bi³⁺ (∼108 pm) [46], which caused the decreased lattice parameters when x = 0.2 (Fig. S3b). When the Cu doping content was above 0.2% mol, the three types of lattice sites of Cu atoms coexisted, but most of the Cu still occupied the interstitial sites, leading to slowly increased lattice parameters.

### Multiple Cu roles for improving carrier mobility

**Figure 3.**
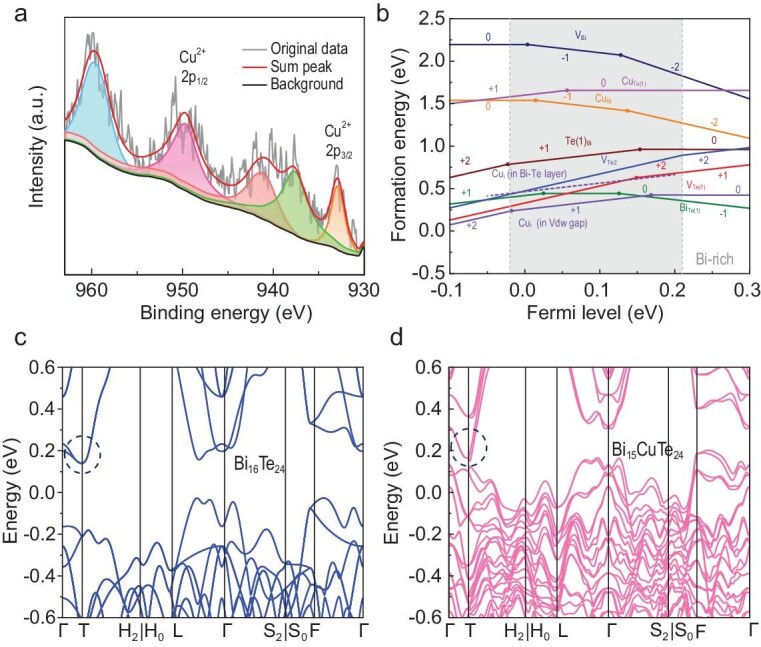
(a) X-ray photoelectron spectroscopy (XPS) spectra of the BTS + 0.2%Cu sample. (b) The calculated defect formation energy of corresponding defects in the Bi_2_Te_3_ + Cu system for the Bi-rich condition by using density functional theory (DFT). (c, d) Calculated electronic band structures of (c) Bi_16_Te_24_ and (d) Bi_15_CuTe_24_.

X-ray photoelectron spectroscopy (XPS) was conducted on the BTS + 0.2%Cu sample to further explore the bonding state of Cu in the matrix, as illustrated in Fig. 3a. The 2p electron binding energy spectrum of Cu exhibited characteristic peaks at ∼933 eV and ∼953 eV and a strong Cu^2+^ satellite peak, consistent with the reported orbital peaks of Cu in the +2 oxidation state [47]. Thus, it can be inferred that the Cu atoms bond with the matrix. To further elucidate the existence form of Cu atoms in the lattice, the defect formation energies of BTS + Cu systems have been investigated as shown in Fig. 3b. Various defect forms were considered, including Cu interstitials in the vdW gaps (Cu_i_ in vdW gap), Cu inside the quintuple layers (Cu_i_ in Bi-Te layer), Cu occupying Bi sites (Cu_Bi_), Cu occupying Te(1) sites (Cu_Te(1)_), and a series of intrinsic defects such as Bi vacancies (V_Bi_) and Te vacancies (V_Te(1)_). N-type Bi_2_Te_3_ is usually in a Bi-rich environment, and it can be observed that the formation energy of Cu_Bi_ was lower than that of Cu_Te(1)_, suggesting that Cu preferentially occupied the cation site as a p-type dopant. With the electron carrier concentration increased (the Fermi level goes deeper into the conduction band), the formation energy of Cu_Bi_ decreased, which confirmed that Cu was indeed more likely to occupy Bi vacancies at higher carrier concentrations. Thus, the reduction of intrinsic Bi vacancies smooths the lattice to achieve lattice plainification, thereby weakening defect scattering and effectively enhancing *μ*.

In addition, we found that Cu replacing Bi in the lattice could regulate the electronic band structure, as revealed by theoretical calculations. Figure 3c and d illustrates the calculated electronic band structures for Bi_16_Te_24_ and Bi_15_CuTe_24_, respectively. Notably, the energy difference between the conduction band minimum at the T and Γ points in Bi_15_CuTe_24_ is ∼0.16 eV, which is higher than the ∼0.06 eV observed in Bi_16_Te_24_. Additionally, analysis of the band structures along the Γ-T and Γ-H_2_|H_0_ directions showed that the CBM at the T point, which originally exhibited two highly degenerated bands, began to experience a band divergence. Therefore, the introduction of Cu led to a reduction in the band extrema *N*_v_, which in turn decreased m* and enhanced *μ*. Moreover, we observed that the CBM at the T point in Bi_15_CuTe_24_ became sharper than that in Bi_16_Te_24_, suggesting that Cu sharpened the conduction band and reduced the single-band effective mass (*m*_b_*), thereby further increasing *μ*. As shown in Fig. S4a and b, the integral of the total density of states near the CBM in Bi_15_CuTe_24_ was substantially reduced compared to Bi_16_Te_24_, which further confirmed that the introduction of Cu reduced the m* and effectively improved *μ*. And, among the Cu-related defects, two types of Cu_i_ demonstrated the lowest formation energy, which proved that Cu was also likely to enter the vdW gaps and the quintuple layers (Fig. 3b). Combined with the XPS results, we could conclude that Cu_i_ can bond with neighboring atoms, thereby creating additional charge transfer pathways that further enhance *μ*.

### Microstructure characterization for Cu-doped BTS

**Figure 4.**
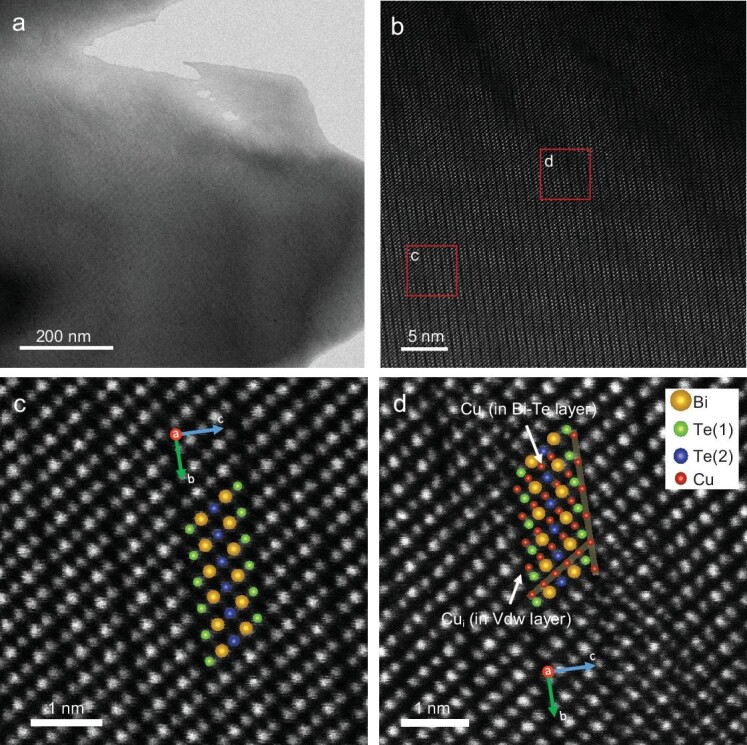
(a) Annular bright field scanning transmission electron microscopy (ABF-STEM) image of the BTS + 0.2%Cu sample. (b) The enlarged high-magnification high-angle annular dark field (HAADF)-STEM image of the BTS + 0.2%Cu sample. (c, d) HAADF-STEM images of the BTS + 0.2%Cu sample, where (c) shows a region of the matrix and (d) shows a region with interstitial Cu.

Subsequently, we characterized the microstructures of the BTS + 0.2%Cu sample using transmission electron microscopy (TEM) to determine the occupation of Cu atoms. Figure 4a displays a low-magnification annular bright field scanning transmission electron microscopy (ABF-STEM) image of the sample, and Fig. S5a–d presents the chemical composition analysis of the corresponding region via energy-dispersive X-ray spectroscopy (EDS). The analysis revealed that there was no Cu-rich precipitate or additional phases in the matrix. Figure 4b displays a high-magnification high-angle annular dark field (HAADF-)STEM image of the sample, showing a distinctive layered structure viewing from [100] zone axis, with further magnification of selected regions shown in Fig. 4c and d. The atomic positions of Bi, Te(Se), and Cu were highlighted by the overlaid Bi_2_(Te, Se)_3_ structural model in the figures. Figure 4c presents a magnified HAADF-STEM image of the BTS matrix region, where Bi is represented by yellow spheres, and Te(1) and Te(2) are depicted as green and blue spheres, respectively. The quintuple layered structure, consisting of -Te(1)-Bi-Te(2)-Bi-Te(1)-, was clearly visible, with these layers interconnected through vdW interactions along -Te(1)-Te(1)- connections. Figure 4d shows a magnified HAADF-STEM image of the Cu-doped region, where Cu atoms are indicated as red spheres, clearly demonstrating that the introduced Cu atoms occupied the vdW gaps and within the quintuple layers, thereby widening the a/c-axis, which was consistent with the obtained lattice parameters (Fig. S3b).

### Thermal transports and *ZT* value

**Figure 5.**
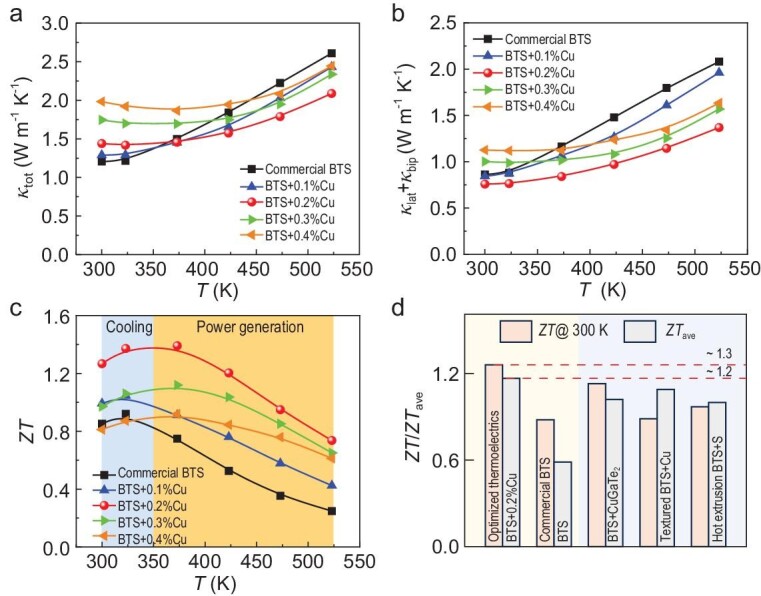
The temperature-dependent (a) total thermal conductivity *κ*_tot_ of BTS + x%Cu samples (x = 0, 0.1, 0.2, 0.3, and 0.4). (b) The sum of lattice thermal conductivity *κ*_lat_ and bipolar thermal conductivity *κ*_bip_ of BTS + x%Cu samples (x = 0, 0.1, 0.2, 0.3, and 0.4). (c) *ZT* values of BTS + x%Cu samples (x = 0, 0.1, 0.2, 0.3, and 0.4). (d) Comparison of the room-temperature *ZT* and average *ZT* (*ZT*_ave_) at 300–523 K between the BTS + 0.2%Cu sample, commercial BTS and other high-performance BTS [35,39,40].

Figure 5a illustrates the total thermal conductivity (*κ*_tot_) of the BTS + x%Cu samples as a function of temperature. The *κ*_tot_ is comprised of electronic (*κ*_ele_), lattice (*κ*_lat_), and bipolar (*κ*_bip_) parts, and Fig. S6a and b shows the relevant parameters that determine the *κ*_tot_, including the thermal diffusivity *D* and specific heat *C*_p_. The increase in room-temperature *σ* led to a gradual enhancement in *κ*_tot_, while at high temperatures, a decreasing trend was observed. The *κ*_ele_ values were calculated using the Wiedemann–Franz law (*κ*_ele_ = *LσT*), where *L* represents the Lorenz number. The *L* and *κ*_ele_ are depicted in Fig. S6c and d. The values of *κ*_lat_ + *κ*_bip_ were obtained by subtracting *κ*_ele_ from *κ*_tot_, as shown in Fig. 5b. Since the intrinsic excitation at room temperature can be neglected, the *κ*_lat_ + *κ*_bip_ values at room temperature were approximately equal to *κ*_lat_. The room-temperature *κ*_lat_ first decreased and then increased with Cu content. Specifically, the sample with x = 0.2 had the lowest *κ*_lat_ of ∼0.76 W m^−1^ K^−1^ at 300 K, which was attributed to the strong point defect scattering for phonons caused by Cu_Bi_ and Cu_i_ atoms. The subsequent increase in *κ*_lat_ might be due to the reduction in defect concentration in the lattice caused by the further introduction of Cu atoms, leading to weakened defect scattering, which was consistent with the continuously increasing *μ* shown in Fig. 2b. Figure 5c shows the variation of *ZT* with temperature, indicating that the *ZT* value of the BTS + 0.2%Cu sample was significantly improved compared to the Cu-free sample at 300–523 K. At 300 K, the optimal *ZT* value reached ∼1.3, with an average *ZT*_ave_ of ∼1.2 (300–523 K), outperforming those of other high-performance n-type BTS thermoelectrics (Fig. 5d) [35,38,40]. Moreover, our high-performance BTS + 0.2%Cu samples also demonstrated exceptional repeatability and thermal stability (Fig. S7), elucidating the promising potential for practical applications.

### Uniformity, mechanical properties, and thermoelectric device performance

**Figure 6.**
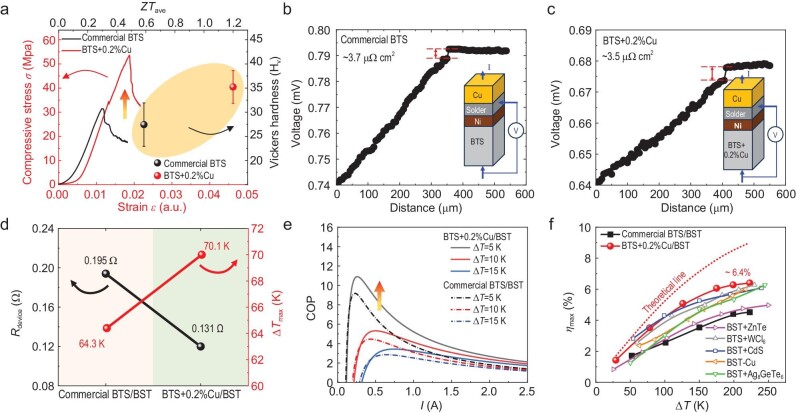
(a) Relationship between the compressive strength, the Vickers hardness, and *ZT*_ave_ of commercial BTS and the BTS + 0.2%Cu sample. (b–c) The measured contact resistance by the four-probe method for (b) across the Cu to commercial BTS interfaces and (c) across the Cu to BTS + 0.2%Cu interfaces. (d) The internal resistance *R*_device_ and maximum cooling temperature difference Δ*T*_max_ of the commercial BTS/BST-based device and BTS + 0.2%Cu/BST-based device. (e) Comparison of the calculated coefficient of performance (COP) for the commercial BTS/BST-based device and BTS + 0.2%Cu/BST-based device at temperature differences Δ*T* of 5 K, 10 K, and 15 K. (f) Maximum conversion efficiency *η*_max_ as a function of temperature difference Δ*T* for the BTS + 0.2%Cu/BST-based device compared with other reported high-performance thermoelectric devices [45,46,48–50]. The red dashed line represents the theoretical *η*_max_ for the BTS + 0.2%Cu/BST-based device.

Furthermore, we characterized the uniformity of the kilogram-scale BTS + 0.2%Cu ingot with mass production on the same horizontal plane. As illustrated in Fig. S8a, we cut the ingot into five areas, excluding the areas with poor crystallinity at the top and bottom of the ingot, and selected line1 and line2 for thermoelectric performance testing. From Fig. S8b–d, it can be seen that the BTS + 0.2%Cu ingots exhibited better thermoelectric performance compared to commercial BTS. Consequently, the BTS + 0.2%Cu ingots, which exhibited highly uniform distribution of thermoelectric properties, provided a robust foundation for the mass production of BTS + 0.2%Cu thermoelectrics. This is essential for subsequent device fabrication and practical applications. Aside from the excellent thermoelectric properties, mechanical performance characterization revealed that the BTS + Cu sample demonstrated superior machinability. As illustrated in Fig. 6a, the compressive strength of the BTS + 0.2%Cu sample achieved ∼54 MPa, exhibiting a notable increase of ∼73% compared to the commercial BTS sample. This improvement in mechanical properties was attributed to the bonding of Cu atoms in the vdW gaps with Te(1) atoms, which enhanced the interlayer bonding strength. It is worth noting that this enhancement not only contributed to the increase in compressive strength but also had a significant effect on the Vickers hardness. As evident from Fig. 6a, the average Vickers hardness exhibited an increasing trend from ∼28.5 H_v_ for the commercial BTS to ∼35.1 H_v_ for the BTS + 0.2%Cu sample, with the latter also possessing a higher *ZT*_ave_ value.

To assess the conversion efficiency of BTS + 0.2%Cu thermoelectrics in cooling and power generation, commercial BST was used as the p-type counterpart to fabricate thermoelectric devices, and the corresponding thermoelectric performance is presented in Fig. S9. Nickel (Ni) was electroplated onto the surfaces of p-/n-type thermoelectrics to serve as the barrier layer. Figure 6b and c illustrates the contact resistivity measurement for Cu-solder-Ni-commercial BTS and Cu-solder-Ni-BTS + 0.2%Cu. After soldering at ∼493 K, it is notable that the contact resistivity measured between the Cu electrode and the thermoelectric element remained ∼3.5 µΩ cm², comparable to the ∼3.7 µΩ cm² of commercial BTS. This low contact resistivity indicated a highly efficient electrical interface, which was crucial for optimizing the conversion efficiency of thermoelectric devices [14,42]. Subsequently, we conducted several thermal cycles on BTS + 0.2%Cu samples with electroplated Ni layers at a temperature of 250°C to simulate long-term device operating conditions. The SEM and EDS characterization indicate that after prolonged thermal cycling, Ni began to infiltrate the substrate to a depth of ∼3.5 μm (Fig. S10), which might potentially deteriorate the interfacial contact resistance and subsequently affect device performance. Therefore, we believe that further optimizing the contact layer and reducing the chemical reaction between the contact layer and the matrix could potentially enhance device performance as well as ensure device reliability and lifetime.

For cooling, the most intuitive parameter to evaluate cooling performance is the maximum cooling temperature (Δ*T*_max_) [41]. In this study, we examined two full-scale 7-pair thermoelectric devices fabricated from n-type commercial BTS and BTS + 0.2%Cu combined with p-type commercial BST, each with dimensions of 10 × 10 × 6 mm³ (Fig. 1b, inset). The measured cooling Δ*T*_max_ and the internal resistance of the devices (*R*_device_) are presented in Fig. 6d and Fig. S11a–d. It can be observed that the BTS + 0.2%Cu/BST device had an excellent Δ*T*_max_ of ∼70.1 K and low *R*_device_ compared to those of the commercial BTS/BST device at the hot-end temperature (*T*_h_) of ∼303 K. Further, the BTS + 0.2%Cu/BST device achieved a higher Δ*T*_max_ of ∼85.6 K at the *T*_h_ of ∼343 K (Fig. S11b), showing considerable application advantages over commercial devices at temperatures above 303 K (Fig. S11d). The calculated coefficient of performance (COP) for the BTS + 0.2%Cu/BST device was better than that of the commercial BTS/BST device (Fig. 6e), suggesting the potential for BTS + 0.2%Cu thermoelectrics to have lower power consumption when used in practical solid-state cooling applications.

To further confirm the high-level *ZT*_ave_ of ∼1.2 (Fig. 5d), we also assessed the power generation efficiency of the two thermoelectric devices (Fig. S12a–f). As illustrated in Fig. S12a and d, as the current (*I*) changed, the open-circuit voltage (*V*) increases progressively with the rising temperature difference Δ*T*. The slope of the *V*-*I* curve indicated the internal resistance of the thermoelectric device. According to Fig. S12a, the internal resistance of the BTS + 0.2%Cu-based device rose from ∼0.143 Ω at a Δ*T* of ∼29 K to ∼0.213 Ω at a Δ*T* of ∼223 K, which was lower than the ∼0.281 Ω of the commercial BTS-based device at a Δ*T* of ∼223 K (Fig. S12d). This further reflected the higher *σ* in BTS + 0.2%Cu. Figure S12b demonstrates that the maximum output power of the BTS + 0.2%Cu-based device was ∼0.42 W at a Δ*T* of ∼223 K, significantly exceeding the ∼0.24 W achieved by the commercial BTS-based device under the same Δ*T* (Fig. S12e). This enhancement could be attributed to the exceptionally high *PF* in BTS + 0.2%Cu in the temperature range of 300–523 K. Additionally, the BTS + 0.2%Cu-based device achieved a maximum conversion efficiency *η*_max_ of ∼6.4% at a Δ*T* of ∼223 K (Fig. S12c), outperforming both the commercial BTS-based device (Fig. S12f) and other Bi_2_Te_3_-based devices (Fig. 6f) [45,46,48–50]. The excellent cooling Δ*T*_max_ and *η*_max_ strongly demonstrated that BTS + 0.2%Cu exhibited superior thermoelectric efficiency in the low-to-mid temperature ranges.

## CONCLUSIONS

In this work, we substantially boosted the carrier mobility in n-type BTS by incorporating trace Cu atoms to realize lattice plainification. The multiple occupations of Cu substantially optimized the electrical performance over the low-to-medium temperature ranges. Specifically, Cu atoms can occupy Bi vacancies, thereby achieving lattice plainification and significantly weakening the defect scattering on carriers. Meanwhile, Cu entering the Bi site was observed to effectively alter the conduction band structure, promoting band divergence and sharpening, which in turn reduced the effective mass and further boosted carrier mobility. Moreover, Cu atoms also occupied interstitial positions in the BTS lattice, which were found to form additional charge transport pathways to facilitate electron transport by bonding with adjacent atoms. As a result, we obtained superior thermoelectric performance over a wide temperature range in the kilogram-scale, mass-production BTS + 0.2%Cu ingots with excellent uniformity and enhanced mechanical properties, which elucidate the great potential for direct applications in both power generation and solid-state cooling. The as-fabricated full-scale thermoelectric power generators and coolers demonstrated advantageous device efficiencies and lower power consumptions compared with the commercially available devices. This study presents a comprehensive investigation into the atomic occupation and role of trace Cu atoms in modulating the thermoelectric properties of n-type BTS. The current results will contribute to the promotion of practical applications in waste heat recovery and electronic cooling with Bi_2_Te_3_-based devices.

## Supplementary Material

nwae448_Supplemental_File
