# Supplementary material for: Lattice plainification and band engineering lead to high thermoelectric cooling and power generation in n-type Bi2Te3 with mass production
Source: Natl Sci Rev. 2024 Dec 6;12(2):nwae448. doi: 10.1093/nsr/nwae448 (PMC11737397; doi:10.1093/nsr/nwae448)
Supplement: nwae448_Supplemental_File [file nwae448_supplemental_file.pdf]

Supporting data

Lattice plainification and band engineering lead to high thermoelectric cooling and power generation in n-type Bi_2Te_3 with mass production

Dongrui Liu[1,2], Shulin Bai[1], Yi Wen[1], Jiayi Peng[1], Shibo Liu[1], Haonan Shi[1], Yichen Li[1], Tao Hong[1], Huiqiang Liang[3], Yongxin Qin[1], Lizhong Su[4], Xin Qian[3], Dongyang Wang[5], Xiang Gao[6], Zhihai Ding[7], Qian Cao[7], Qing Tan[1,*], Bingchao Qin[1,2,*], Li-Dong Zhao[1,2,8,*]

[1]School of Materials Science and Engineering, Beihang University, Beijing 100191, China;
[2]Center for Bioinspired Science and Technology, Hangzhou International Innovation Institute, Beihang University, Hangzhou 311115, China;
[3]Hebei Key Laboratory of Optic-Electronic Information and Materials, College of Physics Science and Technology, Hebei University, Baoding 071002, China;
[4]School of Materials Science and Engineering, Taiyuan University of Science and Technology, Taiyuan 030024, China;
[5]Key Laboratory of Materials Physics of Ministry of Education School of Physics, Zhengzhou University, Zhengzhou 450001, China;
[6]Center for High Pressure Science and Technology Advanced Research (HPSTAR), Beijing 100094, China;
[7]Huabei Cooling Device Co. LTD, Hebei 065400, China;
[8]Tianmushan Laboratory, Yuhang District, Hangzhou 311115, China

***Corresponding authors.** E-mails:
zhaolidong@buaa.edu.cn; qinbingchao@buaa.edu.cn; tanqing@ustb.edu.cn

Experimental and calculation details

Synthesis and ingot growth: We have selected Bi blocks (99.99%, Aladdin element, China), Te blocks 99.99%, Aladdin element, China), Se blocks (99.99%, Aladdin element, China), TeI_4 powder (99.99%, Aladdin element, China), Cu filaments (99.999%, Aladdin element, China) as the raw materials. The stoichiometric ratios of $Bi_2Te_{2.79}Se_{0.21}I_{0.004}$+x%Cu (x = 0, 0.1, 0.2, 0.3, and 0.4) were calculated from the weight and configuration of the high-purity elemental composition of Bi, Te, Se, TeI_4, and Cu. $Bi_2Te_{2.79}Se_{0.21}I_{0.004}$+x%Cu abbreviated as BTS+x%Cu over the entire text were used since all the samples were doped with a fixed content of I. In this study, the commercial BTS refers to BTS+0%Cu. To improve the uniformity of the ingot samples, the mixed raw materials were first sealed in quartz tubes and then placed in a shaking furnace, where they were melted at 1073 K for two hours. Subsequently, the synthesized polycrystalline ingots were subjected to slow crystal growth in a homemade zone melting furnace. Additionally, the commercial p-type $(Bi, Sb)_2 Te_3$ (BST) used in this study was prepared using the same method, and its thermoelectric performance was provided in Figure S9. Finally, BTS+x%Cu ingots with dimensions of roughly ~31 mm in diameter and ~34 cm in length were produced for further performance measurements.

Structural characterization: The phase composition and preferred orientation of the samples were characterized using X-ray diffraction (XRD) with a D/max 2200PC X-ray diffractometer (Rigaku, Japan), utilizing Cu Kα (λ = 1.5418 Å) radiation, operating at 40 kV and 200 mA, and equipped with a position-sensitive detector. The valence states of the elements were determined using X-ray Photoemission Spectroscopy (XPS; Thermo Scientific ESCALAB 250Xi). To prepare samples for transmission electron microscopy (TEM), the specimens were initially cut and polished until a thickness of 20-30 μm was achieved, followed by argon ion milling using a Precision Ion Polishing System Model 695 PIPS Ⅱ (Gatan) at an applied voltage of 3.5-1.0 kV and a low temperature of 120 K. Scanning transmission electron

microscopy (STEM) analysis was conducted using a JEM-ARM200F (JEOL) microscope operating at 200 kV.

Electrical transport properties: The obtained ingots were cut and polished as ~3 × 3 × 8 mm^3 rectangular shaped samples and the electrical conductivity and Seebeck coefficient were measured simultaneously using the Ulvac Riko ZEM-3 and CTA instruments in a helium atmosphere. The test temperature ranges from 300 K to 523 K. The measurement error in the Seebeck coefficient and electrical conductivity was within 5%. Hall coefficient (R_H) was measured by Hall test system (Lake Shore 8400) at a reversible magnetic field (±1T) using the Van der Pauw method at 300 K. The carrier concentration (n) is given by $n = 1/(e \cdot R_H)$ and the carrier mobility (μ) is calculated by the following relation: $\mu = \sigma \cdot R_H$, where σ is the electrical conductivity.

The calculation of deformation potential:

The deformation potential (Ξ) was calculated by [1, 2]:

$$\Xi = \left[9\mu_W \left(\frac{T}{300} \right)^{\frac{5}{2}} T^{-1} \frac{3\pi m_I^*}{2k_B^2 \hbar C_l N_V} \right]^{-\frac{1}{2}} \quad \text{(S1)}$$

where μ_W is the weighted mobility, T is the temperature, m_I^* is the inertial effective mass, k_B is the Boltzmann constant, $\hbar$ is the Reduced Planck constant, C_l longitudinal elasticity modulus calculated by $C_l = v_l^2 \rho$ (v_l is longitudinal sound velocity and ρ is the density) and N_v is orbital degeneracy.

In this work, the relative deformation potential (Ξ/Ξ_0) is calculated as follows:

$$\frac{\Xi}{\Xi_0} = \left(\frac{\mu_W}{\rho v_l^2} \right)^{-\frac{1}{2}} \left(\frac{\rho_0 v_{l0}^2}{\mu_{W0}} \right)^{-\frac{1}{2}} \quad \text{(S2)}$$

where the parameters with 0 as the subscript represent the data of the matrix sample (x = 0).

Thermal transport properties: To measure the thermal conductivity, cylinders with

a diameter of 6 mm and a thickness of ~1 mm for BTS+x%Cu ingots were prepared. To minimize errors in the thermal conductivity measurement, a thin layer of graphite was applied to the sample. Thermal conductivity was determined using the formula $\kappa = D{\cdot}\rho{\cdot}C_p$, where thermal diffusivity (D) was measured with a Netzsch LFA457 instrument employing the laser flash diffusivity method, ρ was the sample density obtained with a gas pycnometer (Micromeritics AccuPyc II 1340), and C_p was the heat capacity derived from the Debye model [3]. The thermal diffusivity data were analyzed using the Cowan model with pulse correction. The uncertainty in the thermal conductivity measurement was estimated to be within ~8%, accounting for all uncertainties in D, ρ, and C_p.

Mechanical performance measurement: The Vickers hardness of the samples was tested using a Vickers hardness tester (HVS-1000TM/LCD) with a test load of 0.5 N and a holding time of 10 seconds. The room-temperature compressive strength of the samples was tested using a small-scale mechanical testing platform. During the compression process, the compression speed was 0.005 mm min^{-1}, and the sample dimensions were ~2 × 2 × 4 mm^3.

The uniformity of thermoelectric performance measurement: The top and bottom ends of the BTS+0.2%Cu ingot, which exhibited poor crystallinity, were trimmed. The remaining ingot was then evenly divided into five segments. Samples for electrical and thermal performance tests were prepared as shown in Figure S8.

Thermoelectric device fabrication and performance measurement: The Mini-PEM from Advance Riko company was used to examine the power generation performance of the 7-pair n-type BTS+0%Cu (commercial BTS)/p-type BST-based and n-type BTS+0.2%Cu/p-type BST-based devices. Commercial BST/BTS-based thermoelectric devices mentioned in this study refers to custom-made devices using commercial BST/BTS-based materials from Huabei Cooling Device Co. LTD. The

n-type and p-type Bi_2Te_3-based thermoelectrics were cut into slices with a thickness of ~6 mm and electroplated with Ni as a contact layer on the upper and lower surfaces. The slices were then cut into ~1.95 × 1.95 × 4.8 mm^3 legs. 7 pairs of n-p couples were soldered with copper aluminum nitride ceramics to assemble a thermoelectric device with a size of 10 × 10 × 6 mm^3. The solder in low-temperature and high-temperature side was both Sn-Ag-Cu alloys. Copper wires were soldered onto the cold side copper electrodes. The electrical output power, output voltage, and the conversion efficiency of the power generation devices were measured using a Mini-PEM testing system. The cold-side temperature (T_c) was set at ~293 K by a water cooler. Theoretical conversion efficiency was simulated using COMSOL® Multiphysics software by setting thermoelectrics dimensions, thermoelectric properties, and other limiting conditions.

Cooling performance measurement of thermoelectric devices: The maximum cooling temperature difference (ΔT_{max}) and internal resistance (R_{device}) of the thermoelectric devices were tested using the Z-Meters (a commercial thermoelectric cooler test equipment, RMT Ltd., Russia). And, the reference temperatures on the Z-Meters testing interface were set to 303 K and 343 K, and then testing began to obtain cooling ΔT_{max} and R_{device}. Theoretical cooling ΔT_{max} was simulated using COMSOL® Multiphysics software by setting thermoelectrics dimensions, thermoelectric properties, and other limiting conditions.

Calculations of coefficient of performance (COP): The COP for a pair of the p-n thermoelectric device is defined as the ratio between the cooling capacity (Q_c) and the electrical power consumption (P). In a pair of the p-n thermoelectric device, the Q_c, the P, and the COP can be expressed as [4]:

$$Q_c = (S_p - S_n)IT_c - \frac{1}{2}I^2R - \kappa(T_h - T_c) \tag{S3}$$

$$P = I^2R + I(S_p - S_n)(T_h - T_c) \tag{S4}$$

$$\text{COP}=\frac{Q_c}{P}=\frac{(S_p-S_n)IT_c-\frac{1}{2}I^2R-\kappa(T_h-T_c)}{I^2R+I(S_p-S_n)(T_h-T_c)} \tag{S5}$$

where S_p, S_n, I, T_c, T_h, R, and κ represent the Seebeck coefficient of the p-leg, the Seebeck coefficient of the n-leg, electrical current, cold-end temperature, hot-end temperature, total electrical resistance, and total thermal conductivity, respectively.

Interfacial contact resistivity measurement and microstructure characterization: The interfacial contact resistance (ρ_c) of all junctions was measured using a home-made four-probe measurement system. The leg with interfacial layers was scanned from thermoelectrics towards Cu electrodes, with the voltage being monitored at a constant electrical current of 100 mA. ρ_c was evaluated based on the abrupt voltage change observed between the thermoelectrics and the Cu electrodes. Furthermore, we subjected n-type Bi_2Te_3-based thermoelectric particles with the Ni layer to a heat treatment cycle, maintaining them at 523 K for 12 hours, and repeated this cycle four times. This procedure simulates the long-term operational conditions of the thermoelectric device, enabling us to investigate the diffusion phenomena occurring between the contact layer and the n-type Bi_2Te_3-based thermoelectrics.

Density functional theory calculations: First-principles calculations with projected augmented wave (PAW) pseudopotential formalism were performed within the Perdew-Burke-Ernzerhof (PBE) exchange-correlation functional form of generalized gradient approximation (GGA) method as implemented in Vienna *Ab-initio* Simulation Package (VASP) software [5-7]. The electronic structures were calculated based on $Bi_{16}Te_{24}$ and $Bi_{15}CuTe_{24}$ supercells. The wave functions were adopted in plane wave basis with the kinetic energy cut-off of 550 eV, and the spin-orbital coupling (SOC) [8] was also adopted. The convergence criterions for the total energy and Hellmann-Feynman force were less than 10^{-8} eV and 10^{-2} eV/Å, respectively.

For conducting defect calculations, the 3 × 3 × 1 supercells containing 135 atoms for the Bi_2Te_3 were performed in this work, and the Monkhorst-Pack ***k***-meshes of 4 ×

4 × 2 was used by the conjugated gradient method to sample in the Brillouin Zone (BZ). The wave functions were adopted in plane wave basis with the kinetic energy cut-off of 450 eV. The SOC were also separately considered in our self-consistent calculations [8], which would affect the bandgaps and the positions of the band edges. The convergence criterions for the total energy and Hellmann-Feynman force were less than 10^{-6} eV and 10^{-2} eV/Å, respectively. The lattice constant and ion position of the perfect supercell were relaxed, while for the supercells containing defects, the cell volumes were kept constant and the ion positions were relaxed so as to meet the dilute limit condition [9, 10].

The stability of a defect was determined by its formation energy, which is defined as [10]:

$$\Delta H_{D,q} = E_{D,q} - E_{\text{perfect}} - \sum_i n_i \mu_i + q(E_{\text{V}} + E_{\text{F}} + \Delta V) \tag{S6}$$

where $\Delta H_{D,q}$ represents the formation energy of a defect (*d*) in charge state (*q*). $E_{D,q}$ and E_{perfect} are the total energy of the defect system and perfect supercell, respectively. n_{i} and E_{i} are the number and total energy of the *i*-th type (host atoms or impurity atoms) added to ($n_i > 0$) or taken from ($n_i < 0$) the supercells in order to create the defect. μ_i refers to the chemical potential of an *i* atom with respect to that of an elemental phase (μ_i^{el}). E_{F} is the Fermi level with respect to E_V, which is the valence band maximum (VBM) of the perfect supercell. ΔV indicates the electrostatic potential difference between the perfect supercell and defective system, which is aligned with the corresponding VBM. The difference of the average electrostatic potential with the atoms far away from the defect sites was adopted in this work [10].

The chemical potential of the elements determines the growth environment of the crystal. To evaluate the desired and avoid undesired defects, the growth conditions must be chosen carefully. Therefore, the chemical potential of the elements in this work can be defined as:

$$\Delta\mu_{\mathrm{Bi}} = \mu_{\mathrm{Bi}} - \mu_{\mathrm{Bi}}^{\mathrm{el}}, \quad \Delta\mu_{\mathrm{Te}} = \mu_{\mathrm{Te}} - \mu_{\mathrm{Te}}^{\mathrm{el}} \tag{S7}$$

Meanwhile, it must meet:

(1) The compound can exist stably:

$$2\Delta\mu_{\mathrm{Bi}} + 3\Delta\mu_{\mathrm{T}e} = \Delta H_f(\mathrm{Bi_2Te_3}) \tag{S8}$$

(2) All component elements should be beneficial to the formation of compounds, not pure phase elements:

$$\Delta\mu_{\mathrm{Bi}} < 0, \quad \Delta\mu_{\mathrm{Te}} < 0 \tag{S9}$$

Among them, $\Delta H_f(\mathrm{Bi_2Te_3})$ represents the enthalpy of formation for the Bi_2Te_3 (mp-34202) structure based on Materials Project [11], respectively. Based on the previously mentioned relationships, it can be determined that, under Te-rich (Bi-rich) condition, the permissible upper limits for the chemical potentials of Te and Bi are -0.272 (0) and 0 (-0.272) eV in the Bi_2Te_3, respectively.

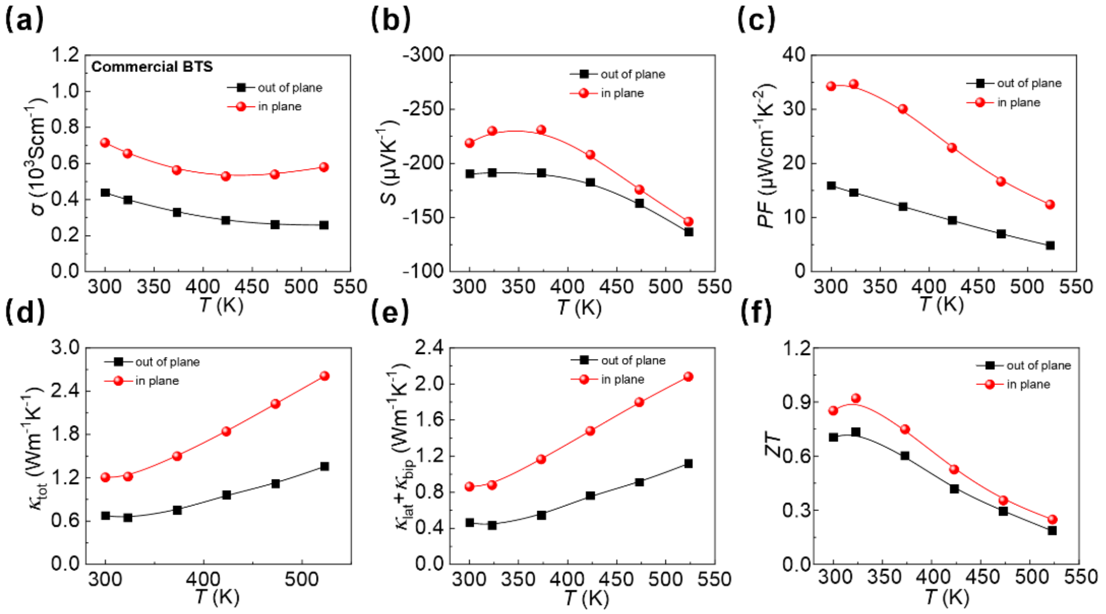

Figure S1. Thermoelectric performance of the commercial BTS sample along the in-plane and out-of-plane directions. (a) Electrical conductivity (σ). (b) Seebeck coefficient (S). (c) Power factor (PF). (d) Total thermal conductivity (κ_{tot}). (e) The sum of lattice thermal conductivity (κ_{lat}) and bipolar thermal conductivity (κ_{bip}). (f) Dimensionless figure of merit (ZT).

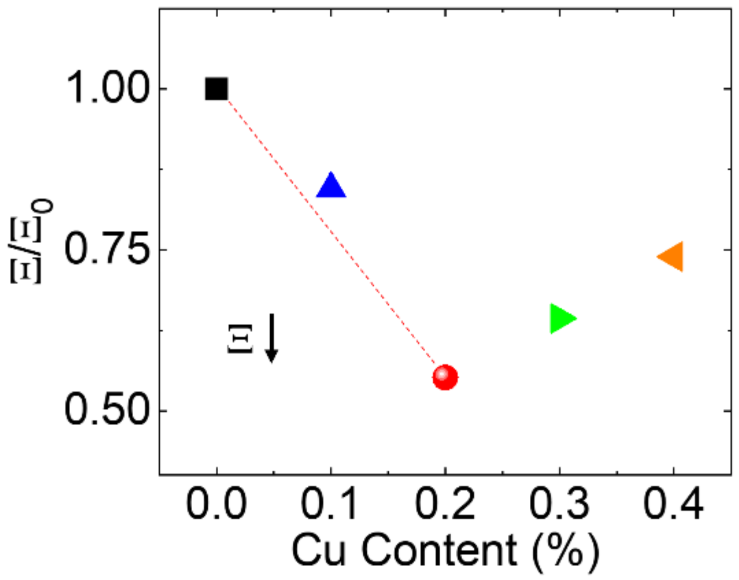

Figure S2. The calculated relative deformation potential (Ξ/Ξ_0) as functions of Cu content for BTS+Cu samples in this work.

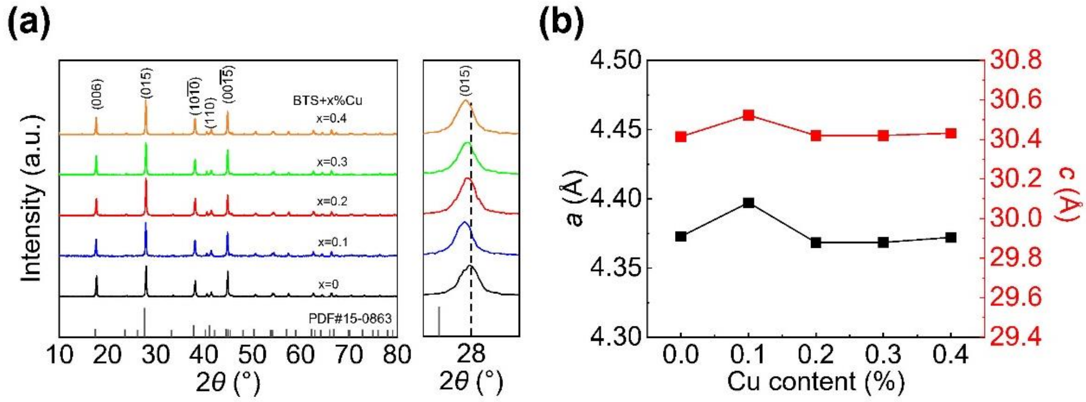

Figure S3. (a) Powder X-ray diffraction (XRD) patterns of BTS+x%Cu samples at room temperature. (b) The lattice parameters of the *a*-axis and *c*-axis as a functions of Cu content.

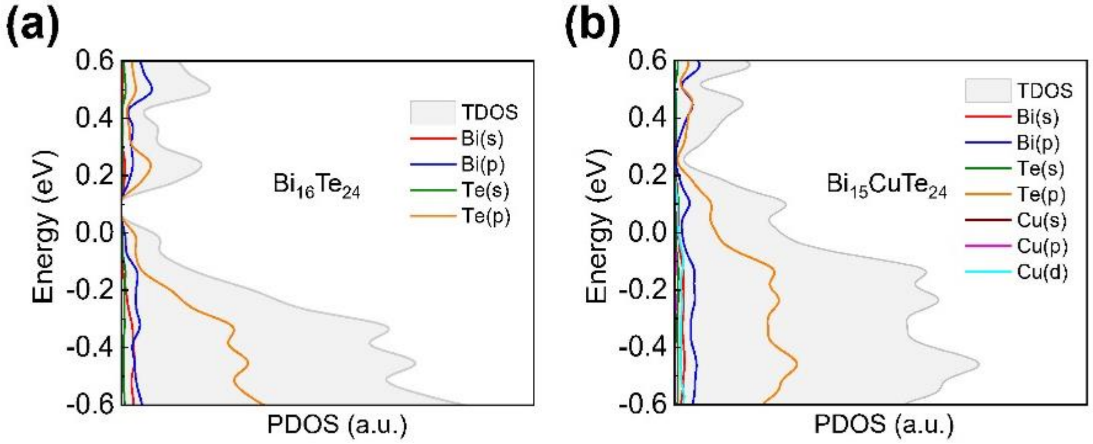

Figure S4. Calculated electronic band structure of (a) $Bi_{16}Te_{24}$ and (b) $Bi_{15}CuTe_{24}$ correspond to the projected density of state (PDOS).

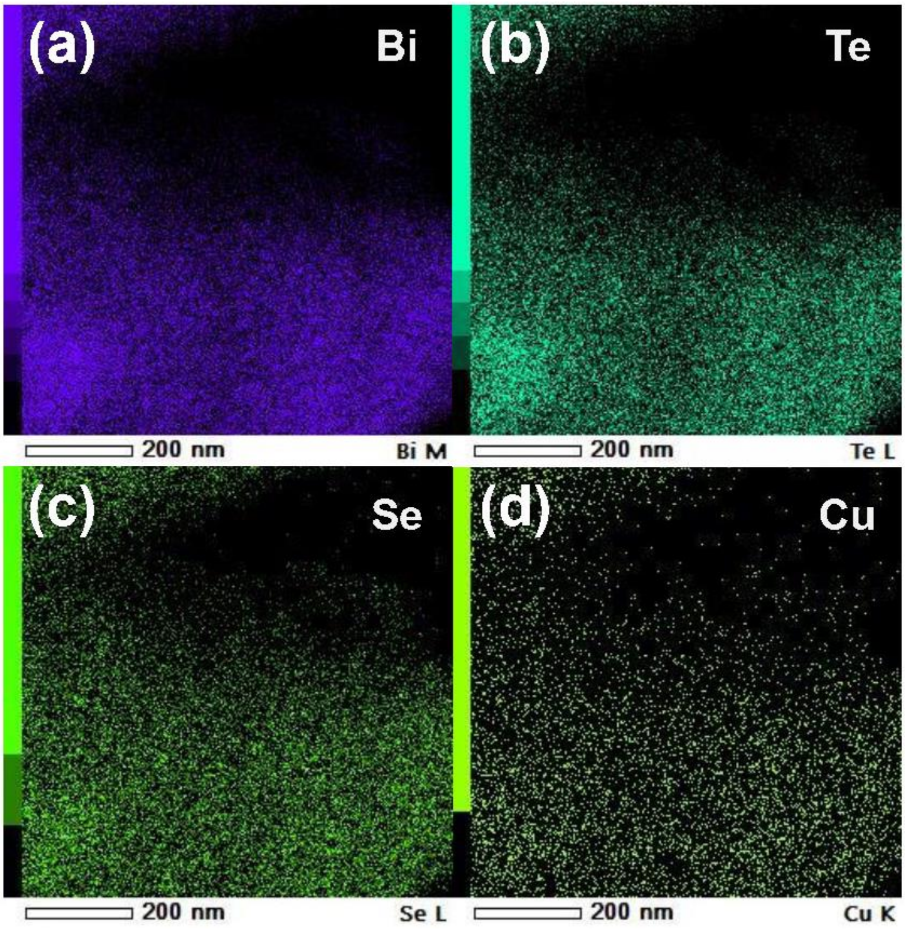

Figure S5. (a-d) Energy dispersive spectroscopy (EDS) element mapping of Bi, Te, Se, and Cu of corresponding region in Figure 4a.

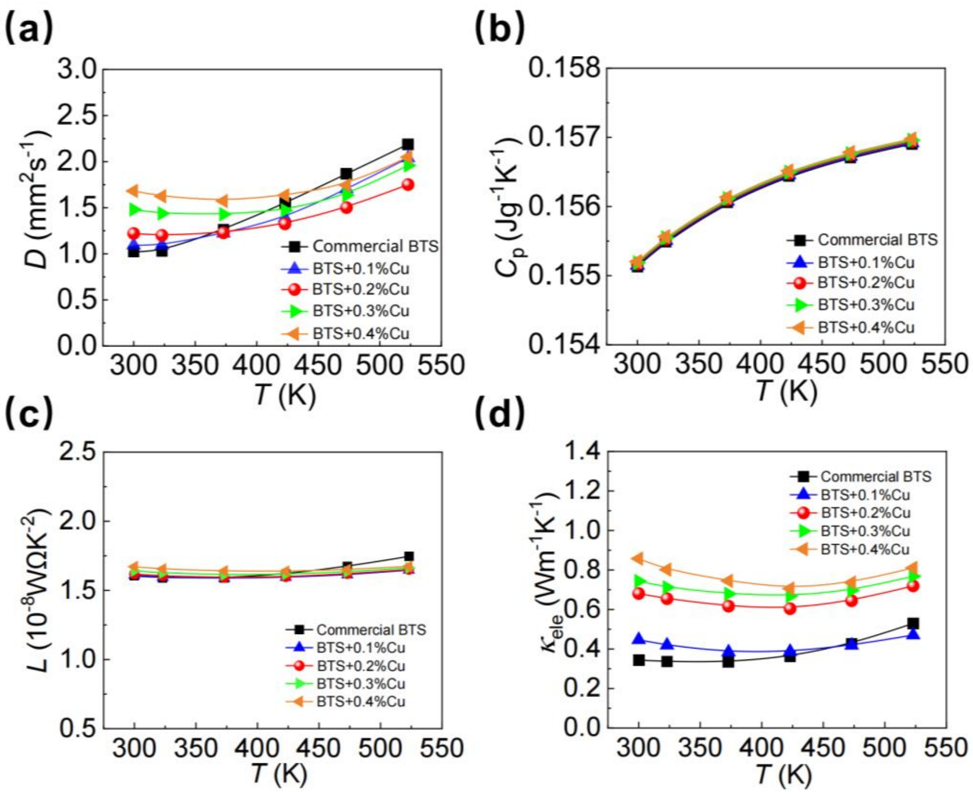

Figure S6. The thermal transport properties as a function of temperature for BTS+x%Cu samples. (a) Thermal diffusivity D. (b) Heat capacity C_p. (c) Lorenz number L. (d) Electronic thermal conductivity κ_{ele}.

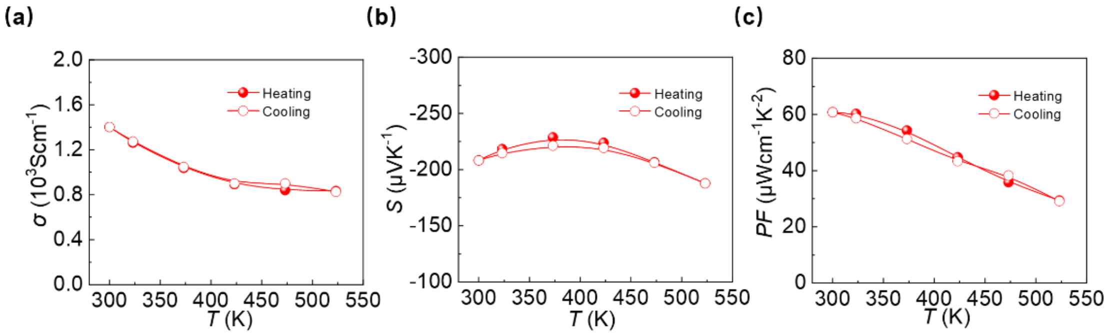

Figure S7. The electrical performance cycle curves of the BTS+0.2%Cu sample. (a) Electrical conductivity (σ). (b) Seebeck coefficient (S). (c) Power factor (PF).

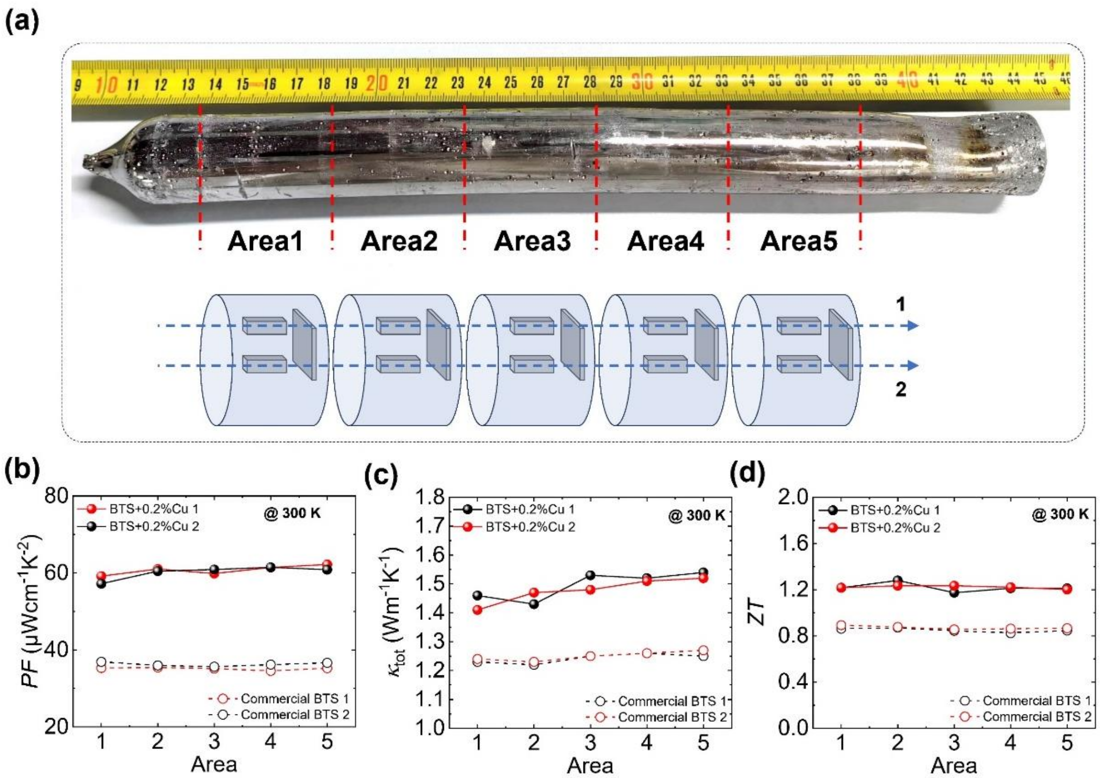

Figure S8. (a) Diagram of commercial BTS and BTS+0.2%Cu ingot uniformity test area and (b-d) the corresponding thermoelectric performance test results along line 1 and line 2 at 300 K: (b) Power factor *PF*, (c) Total thermal conductivity κ_{tot}, and (d) *ZT* values.

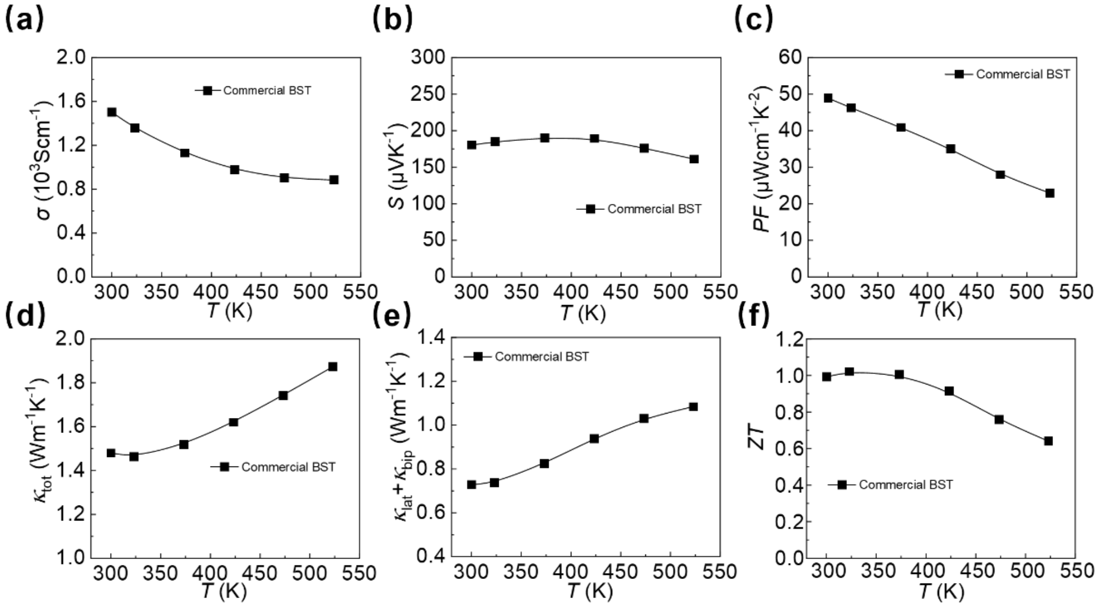

Figure S9. Thermoelectric performance for the used p-type commercial BST. (a) Electrical conductivity (σ). (b) Seebeck coefficient (S). (c) Power factor (PF). (d) Total thermal conductivity (κ_{tot}). (e) The sum of lattice thermal conductivity κ_{lat} and bipolar thermal conductivity κ_{bip}. (f) Dimensionless figure of merit (ZT).

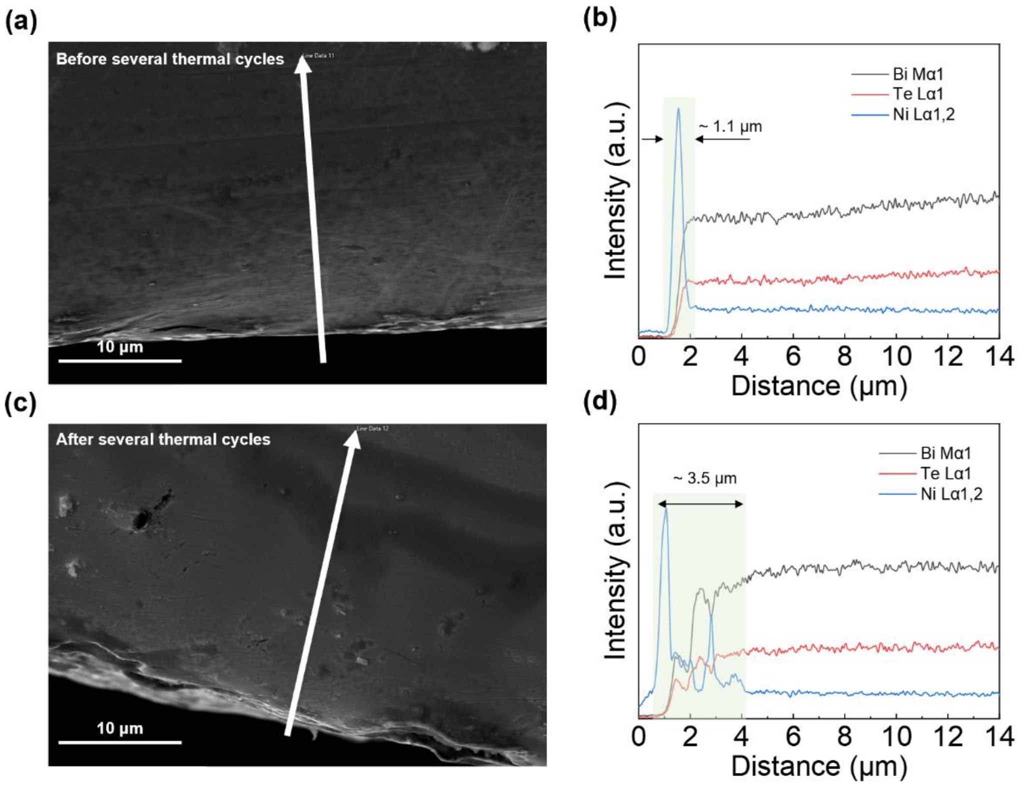

Figure S10. The scanning electron microscope (SEM) images and EDS line scan (indicated by arrow) of BTS+0.2%Cu-Ni surface. (a-b) Before thermal cycle. (c-d) After several thermal cycles.

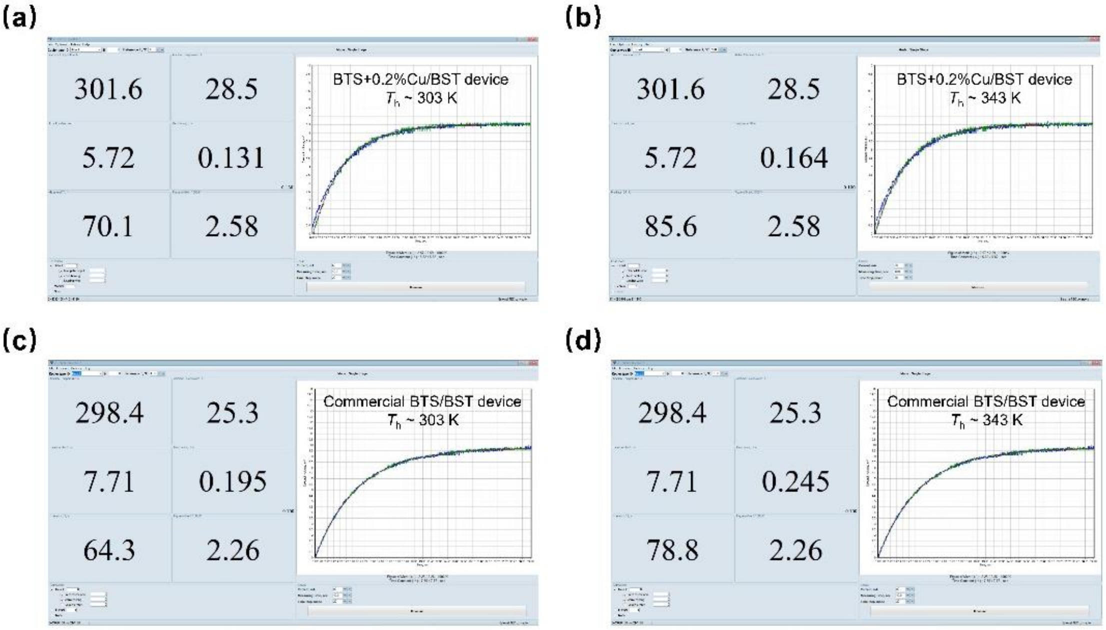

Figure S11. Maximum cooling temperature difference (ΔT_{max}) for the 7-pair BTS+0.2%Cu/BST-based device at the hot-end temperature (T_h) of (a) ~303 K and (b) ~343 K. And, the ΔT_{max} for the 7-pair commercial BTS/BST-based device at the T_h of (c) ~303 K and (d) ~343 K.

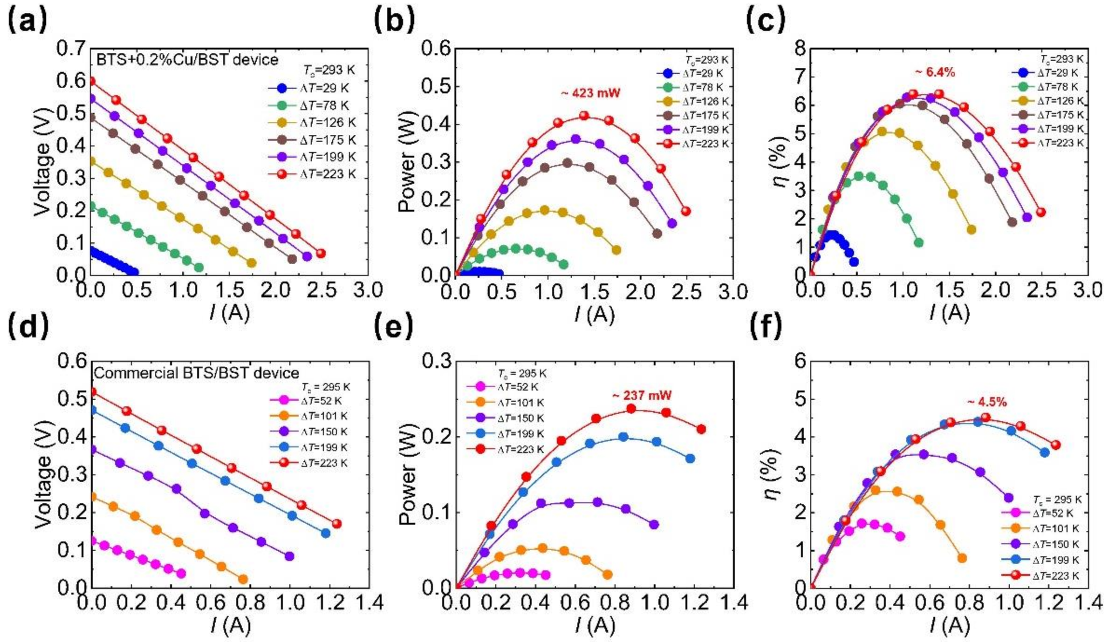

Figure S12. Power generation measurements of the 7-pair BTS+0.2%Cu/BST-based device: (a) Output voltage, (b) Output power, and (c) Conversion efficiency (η) as a function of external current (I) at various temperature differences (ΔT). And, power generation measurements of the 7-pair commercial BTS/BST-based device: (d) Output voltage, (e) Output power, and (f) η as a function of I at various ΔT.

Reference

1. Su L, Wang D, Wang S *et al.* High thermoelectric performance realized through manipulating layered phonon-electron decoupling. *Science* 2022; **375**: 1385-9.
2. Bardeen J, Shockley W. Deformation potentials and mobilities in non-polar crystals. *Phys Rev* 1950; **80**: 72-80.
3. Qin B, Zhang Y, Wang D *et al.* Ultrahigh average *ZT* realized in p-type SnSe crystalline thermoelectrics through producing extrinsic vacancies. *J Am Chem Soc* 2020; **142**: 5901-9.
4. Qin Y, Qin B, Hong T *et al.* Grid-plainification enables medium-temperature PbSe thermoelectrics to cool better than Bi_2Te_3. *Science* 2024; **383**: 1204-9.
5. Blochl P. Projector augmented-wave method. *Phys Rev B Condens Matter* 1994; **50**: 17953-79.
6. Perdew J, Burke K, Ernzerhof M. Generalized gradient approximation made simple. *Phys Rev Lett* 1996; **77**: 3865-8.
7. Hafner J. Ab‐initiosimulations of materials using VASP: density‐functional theory and beyond. *J Comput Chem* 2008; **29**: 2044-78.
8. Filatov M, Cremer D. Calculation of indirect nuclear spin–spin coupling constants within the regular approximation for relativistic effects. *JCP* 2004; **120**: 11407-22.
9. Vidal J, Lany S, d'Avezac M *et al.* Band-structure, optical properties, and defect physics of the photovoltaic semiconductor SnS. *Appl Phys Lett* 2012; **100**: 032104.
10. Walle C, Neugebauer J. First-principles calculations for defects and impurities: applications to III-nitrides. *J Appl Phys* 2004; **95**: 3851-79.
11. Jain A, Ong S, Hautier G *et al.* Commentary: The Materials Project: A materials genome approach to accelerating materials innovation. *APL Mater* 2013; **1**: 011002.
